# Ultrastructural 3D Microscopy for Biomedicine: Principles, Applications, and Perspectives

**DOI:** 10.32607/actanaturae.27323

**Published:** 2024

**Authors:** K. E. Mochalov, D. S. Korzhov, A. V. Altunina, O. I. Agapova, V. A. Oleinikov

**Affiliations:** Shemyakin–Ovchinnikov Institute of Bioorganic Chemistry, Russian Academy of Sciences, Moscow, 117997 Russian Federation; National Research Nuclear University MEPhI (Moscow Engineering Physics Institute), Moscow, 115409 Russian Federation; Moscow Institute of Physics and Technology (National Research University), Dolgoprudny, Moscow Region, 141701 Russian Federation; Academician V.I. Shumakov National Medical Research Center of Transplantology and Artificial Organs, Ministry of Health of the Russian Federation, Moscow, 123182 Russian Federation

**Keywords:** ultrastructural 3D microscopy, electron microscopy, tomography, super-resolution optical microscopy, scanning probe microscopy, biomedical research

## Abstract

Modern biomedical research often requires a three-dimensional microscopic analysis of the ultrastructure of biological objects and materials. Conceptual technical and methodological solutions for three-dimensional structure reconstruction are needed to improve the conventional optical, electron, and probe microscopy methods, which to begin with allow one to obtain two-dimensional images and data. This review discusses the principles and potential applications of such techniques as serial section transmission electron microscopy; techniques based on scanning electron microscopy (SEM) (array tomography, focused ion beam SEM, and serial block-face SEM). 3D analysis techniques based on modern super-resolution optical microscopy methods are described (stochastic optical reconstruction microscopy and stimulated emission depletion microscopy), as well as ultrastructural 3D microscopy methods based on scanning probe microscopy and the feasibility of combining them with optical techniques. A comparative analysis of the advantages and shortcomings of the discussed approaches is performed.

## INTRODUCTION

Methods for the three-dimensional nanoscale analysis of the spectral, morphological, and optical properties of nanostructured specimens need to be improved or elaborated to conduct modern research into biological objects and nanomaterials.

Techniques based on three fundamental microscopy techniques have conventionally been employed for 2D visualization: electron microscopy (EM) [1], optical microscopy (OM) [2], and scanning probe microscopy (SPM) [3]. These approaches have a rather broad scope of application but differ in their key characteristics and provide different types of information about the investigated objects. Thus, EM and SPM allow one to record images with a spatial resolution of several nanometers, making it possible to identify various components of the internal cell structure. The significant drawbacks of these methods include the small field of view and the infeasibility of obtaining information about specimen composition. In contrast, the use of highly specific immunostaining (including multicolor immunostaining) in OM allows for a specific reconstruction of the spatial distribution of the objects of interest (e.g., membrane proteins responsible for any cell–cell interaction process). In many cases, OM can be employed to work with living tissues. Diffraction-limited resolution is the main shortcoming of this method. The Abbe criterion d > λ/2n suggests that a high spatial resolution is achieved at small wavelengths and high refractive index of the medium. An axial (Z-axis) resolution of up to 500 nm and up to 250 nm in the lateral (XY) plane can be achieved when using confocal optical microscopy. Nevertheless, this limitation can be obviated by modern computer processing software and the use of multibeam illumination, which underlies such methods as stochastic optical reconstruction microscopy (STORM) [4] and stimulated emission depletion (STED) microscopy [5].

Transition to three-dimensional analysis is the next stage in developing the aforementioned techniques, since the features of the 3D organization of biological objects and materials have a crucial impact on their functional activity and biological properties in most cases. For this reason, a number of studies focusing on different approaches to solving this problem have recently been published. Therefore, this review aimed to summarize the latest achievements in obtaining three-dimensional images with a nanoscale resolution.

## EM-BASED ULTRASTRUCTURAL 3D MICROSCOPY METHODS

**Fig. 1:**
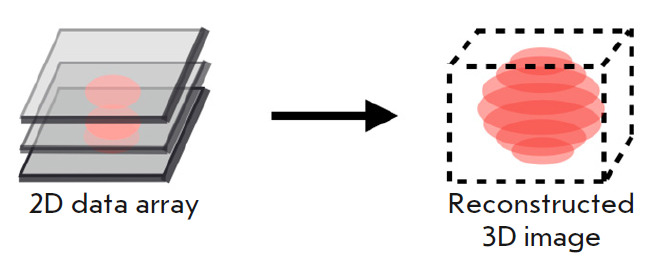
The fundamental principle applied in obtaining three-dimensional images

The key electron microscopy methods include scanning electron microscopy (SEM) and transmission electron microscopy (TEM); each of them has been modified in its own way to obtain 3D images. Acquisition of these images relies on obtaining multiple 2D images of a specimen’s cross sections and merging them into a single 3D image (*Fig. 1*).

**Fig. 2:**
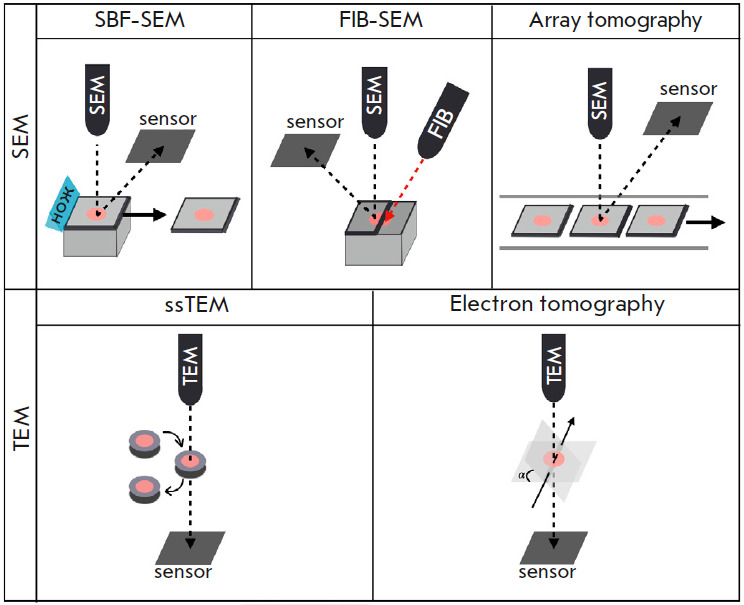
Methods for obtaining three-dimensional images using TEM and SEM. Regardless of the type of microscopy, the first step is specimen preparation, which involves using polymer media to immobilize the specimen and then cut it into sections. Among specimen immobilization methods, the most popular techniques include fixation using methacrylate [8], cryofixation, freeze-fixation, and Tokuyasu cryosectioning [9]. The next step involves accumulating an array of 2D images using TEM or SEM. The recorded images should be aligned with the XY axes and potential rotation should be eliminated, being especially important for systems that study individual sections (ssTEM and array tomography). Next, the area that will be shown in the final image is selected from the resulting array. The final step is creating a full-fledged 3D image from the recorded 2D segments using specialized software

*Figure 2* shows the most advanced 3D image acquisition methods employing this approach. The key differences determining which approach will be selected for a particular task [6] include the method used for creating the serial section images [7], the volume being analyzed, and the maximum spatial resolution.

**Fig. 3:**
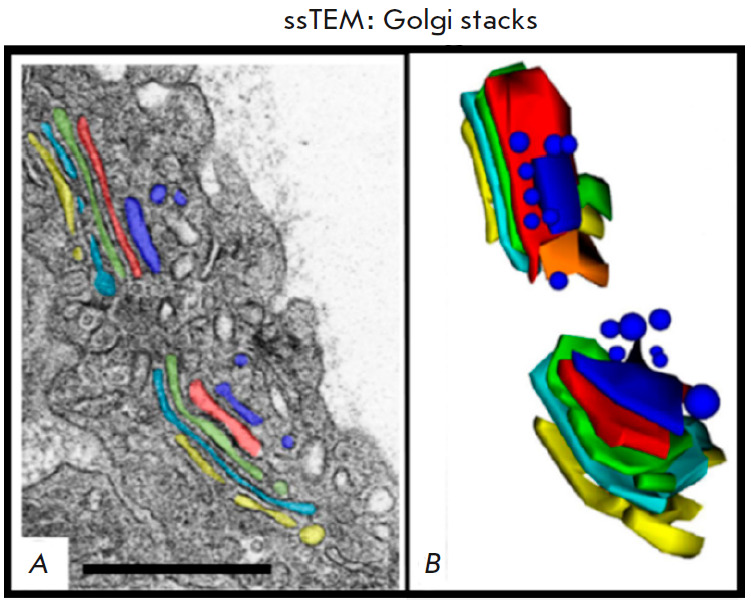
An ssTEM image of Golgi stacks in the mouse lung cell AE1. (*A*) One of the EM microphotographs of the series with color manual segmentation. (*B*) A 3D model of Golgi stacks obtained in TEM based on nine consecutive sections with manually segmented tanks of both stacks. Scale range: 500 nm. The figure was taken from ref. [6]

Serial sectioning TEM (ssTEM) [10], which is TEM involving serial examination of thin sections cut on an ultramicrotome, was a pioneering EM-based 3D microscopy method. The key advantage of ssTEM is that the specimen examination depth is not limited. This method has been pushed to the limit of its capabilities and is intensively employed in a large number of studies [11, 12]. *Figure 3* shows an example of the use of this method.

The main shortcomings of ssTEM involve its labor intensity [13] (as a large number of sections need to be fabricated and examined individually) and technical complexity. The sections have numerous artifacts: mechanical impurities [14], holes, cracks, compressions [15], folds, and uneven the thickness of the section or carrier film [16], which can render adequate data processing arduous. An even bigger problem is the possibility of losing part of the sections due to folds or damage [17, 18]. In such cases, the recorded image can be incorrect and requires separate efforts to eliminate losses.

**Fig. 4:**
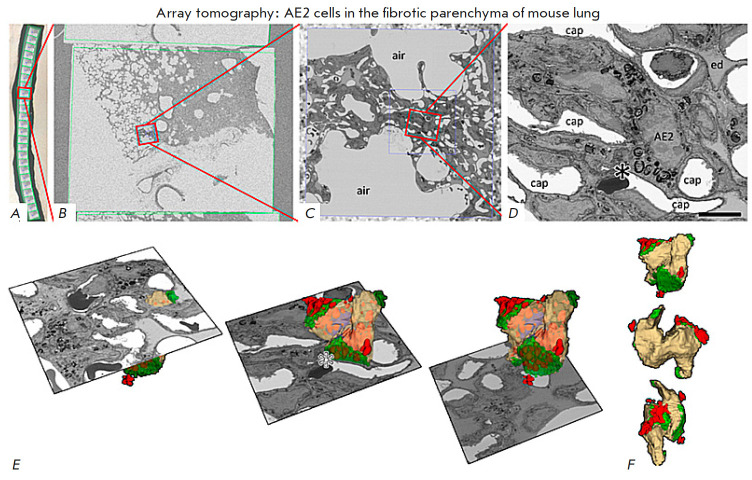
An AT image of an AE2 cell in the fibrotic parenchyma of the mouse lung. (*A*–*D*) Sequential magnification of the SEM image from one of the sections of the tape (*A*). The asterisk on (*D*) corresponds to one AE2 cell in the fibrotic tissue area. Scale bar: 5 μm. (*E*) 3D reconstruction of the AE2 cell. Three separate cross-sectional planes from the sequence of recorded images are shown. (*F*) 3D reconstruction of the AE2 cell at different tilts. The figure was borrowed from ref. [6]

Array tomography (AT) is similar to ssTEM in many respects but employs SEM instead of TEM. This change has made it possible to use microscope slides, silicon wafers, and conductive coating coverslips as substrates [19, 20]. Unlike SEM grids, these substrates have a manifold larger size, and thus allow one to collect more sections. *Figure 4* shows an example of the use of this method.

A special tape can also be used as a substrate, enabling automated acquisition of thousands of sections for a single specimen. This approach is known as automated tape collecting ultramicrotome SEM (ATUM-SEM) [21]. Sections are obtained using an ultramicrotome equipped with a special UMT knife and a water bath. Fragments floating in water are collected by a moving tape, which can be coiled up into a spool if desired. The tape is then cut into pieces, attached to silicon wafers, and SEM images are recorded [19]. The noteworthy advantage of this technique is that post-staining can be used to enhance contrast [22]. Among all the EM techniques, ATUM-SEM boasts the largest field of view, limited only by the width of the ultramicrotome knife. The shortcomings of this technique are generally the same as those for ssTEM; however, they are not as significant because of larger arrays and automated section collection.

**Fig. 5:**
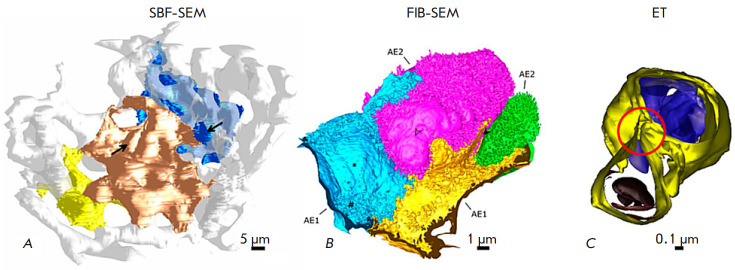
3D reconstructions obtained using serial block-face scanning electron microscopy (SBF-SEM), SEM with layer- by-layer etching with a focused ion beam (FIB-SEM), and electron tomography (ET). (*A*) 3D reconstruction of human alveolar epithelial type 1 cells (AE1) (yellow, gold, and blue) and the alveolar capillary network (white) based on the SBFSEM dataset. Arrows indicate the position of the nuclei of AE1 cells. (*B*) 3D reconstruction of virtually the entire human alveolar epithelial type 2 (AE2) cell (pink) with portions of adjacent AE1 cell domains (blue and yellow) and an additional AE2 cell (green) based on the FIB-SEM dataset. (*C*) 3D reconstruction of the lamellar body (top) and the autophagosome (bottom) inside an AE2 cell (mouse lung) based on the ET dataset. Separate lipid membranes are distinguishable, which in this case is indicative of a connection of two organelles (a red circle). The figure was taken from ref. [6]

SEM images can be obtained not only from sections, but also from the surface of the remaining specimen portion, which underlies the technique known as serial block-face scanning electron microscopy (SBF-SEM). The upper portion of the resin-filled specimen is removed, and the remaining surface is scanned by SEM. This approach eliminates problems such as position alignment, distortion, compression, and section damage. Meanwhile, a problem with this approach is that the charge accumulates in the insulating resin, thus negatively affecting image quality. Additional metallization is required for this reason. When using a high-intensity electron beam, there is a risk of damaging the polymer, which reduces the resolution and plasticity needed for cutting a goodquality surface section [23, 24]. Furthermore, poststaining cannot be used in this approach; so, the original specimen must be characterized by good contrast and conductivity. *Figure 5A* shows an example of the use of this method.

A focused ion beam can be applied for surface sectioning. This approach is known as focused ion beam scanning electron microscopy (FIB-SEM). In all other aspects, the principle of workstation operation is similar to that for SBF-SEM. Structurally, a focused ion beam scanning electron microscope consists of two radiation sources: the top-mounted electron optics for the scanning beam and the side-mounted ion optics for the cutting beam. Both beams focus at a single point, and the entire specimen volume is therefore continuously scanned [25]. Depending on the ion beam current, it is possible to either finely cut a small area or coarsely cut large layers. This method allows one to increase axial resolution from 20 nm for ultramicrotomy to 5 nm for FIB-SEM. Lateral resolution has to be sacrificed for such an increase in axial resolution, since refocusing is only feasible in a region several tens of micrometers in size. Another advantage of ion beam is that it allows one to cut into harder and unstable materials, as well as specimens not embedded in resin. FIB-SEM has already been used for detailed examination of subcellular structures and has demonstrated a high degree of detail of images [26, 27]. The result of this study can be seen in *Fig. 5B*.

Electron tomography (ET) is another three-dimensional- analysis technology [28]. In contrast to all the techniques described above, ET provides virtual, rather than physical, sections of a specimen. The specimen preparation method is similar to that used in TEM and SEM; it involves chemical fixation or cryofixation, and staining or antibody labeling [29]. Therefore, any investigation takes place both at room temperature for specimens embedded in resin and at a cryotemperature for frozen specimens. Below, special attention will be devoted to the aspects of using ET at cryogenic temperatures, which has given rise to a separate technique called electron cryotomography (ECT) [30]. It is also possible to study suspensions of particles smaller than hundreds of nanometers (e.g., viruses, organelles, and macromolecules) by applying them onto a carbon film or a layer of frozen water [31].

In terms of its operation principle, ET is similar to computed tomography, where radiation is directed at different angles, and virtual sections are created. The difference between these two techniques is that an X-ray tube and a camera rotate around the specimen in CT, while the specimen rotates and the camera remains stationary in ET. Because of this design modification, an image is recorded not 360°, but only at 70° with respect to the normal to the specimen at the initial position. This limitation has been termed the “missing wedge” [32] or “missing cone” in the case of using multiple tilt directions [33]. A significantly higher resolution is achieved using this method compared to other EM-based 3D analysis approaches, but it is highly dependent on parameters such as object thickness, accelerating voltage, goniometer tilt calibration, and alignment of the acquired images. The need to achieve a minimum signal- to-noise ratio that suffers from inelastic scattering imposes limitations on the possible specimen size. Thus, accelerating voltages of 200–300 kV are conventionally applied in ET, which allows one to examine specimens up to 300 nm in size. Thicker specimens reduce the achievable resolution [34]. This limitation can be partially obviated either by using the energy filtering approach [35] or by increasing the accelerating voltage. Thus, Vanhecke et al. [36] demonstrated that application of a 400 kV accelerating voltage enabled the examination of a 1 μm thick specimen. Because of the limitations put on the size of the region being investigated, this method is best suited to the study of subcellular structures smaller than 100 nm (*Fig. 5C*). Thicker structures can be examined by ET when making a series of sections and stacking them as it was done in previous methods [27, 37]. Despite the aforementioned limitations, ET, and ECT in particular, produce an absolutely recordbreaking performance for all 3D microscopy types in terms of the achieved spatial resolution. Thus, the record-breaking value of the spatial resolution of 3D reconstruction (2.8 Å) was achieved using the subtomogram averaging (STA) procedure; however, for it to be used, there needs to be a large number of identical nanoscale objects (e.g., protein molecules) available for averaging [30].

Nevertheless, the widespread implementation of ET and ECT remains substantially hampered by their considerable complexity; so, these methods are beingdeveloped and used by only a few research groups [28, 30, 38, 39, 40].

## ULTRASTRUCTURAL 3D MICROSCOPY METHODS BASED ON SUPER-RESOLUTION OPTICAL MICROSCOPY

Optical imaging of biological structures has conventionally been performed using confocal microspectrometry [41], as well as its modifications such as 4Pi microscopy [42, 43, 44]. The main shortcoming of all these techniques is that they come with low spatial resolution. For this reason, various super-resolution optical microscopy methods have been developed over the past two decades and adapted for 3D analysis; the most prominent of those are stochastic optical reconstruction microscopy (STORM) [4] and stimulated emission depletion microscopy (STED) [5]. The practical importance of these techniques is that they enable not only a nanometer resolution structural analysis of specimens, but also the reconstruction of the spatial distribution of target objects thanks to highly specific fluorescent immunostaining, which is an integral step in specimen preparation in any super-resolution OM method.

3D-STORM is the first super-resolution 3D microscopy technique [45]. It belongs to the single-molecule localization microscopy (SMLM) class. This group of techniques relies on the fact that certain fluorophores spontaneously “light up” and “go down,” which can be detected by fast frame-by-frame imaging. In this way, the emission of different subsets of fluorescent molecules will be caught in each frame. The resulting images are still diffraction-limited; however, the positions of individual molecules are determined with a high accuracy from multiple frames using a special mathematical apparatus employing the Delaunay triangulation [46]. The final 3D image is reconstructed using the data obtained by a statistical analysis of thousands of localizations.

Myosin was the first single molecule spatially localized by STORM [47]. For recording images, the molecules (fluorescent probes) were rarefied to such an extent that their signals did not overlap. In this way, it became possible to find the positions of each probe with great accuracy. A fundamentally different approach employing photoswitchable cyanine dyes was developed in ref. [48]. Measurements were conducted as follows: low-intensity radiation switched on a small number of probes; the image was then recorded and probe positions were calculated; radiation with a different wavelength switched this set of probes off and switched another one on. This procedure was repeated many times, yielding a complete 3D image of the specimen. The spatial resolution achieved using this method ranges from 20 to 30 nm. Photo-activated light microscopy is a variation of STORM; it is based on a similar approach, but photoactivated proteins are used instead of dyes [49]. In general, the differences in SMLM techniques also come down to the fluorophores used for specific labeling. For example, the direct STORM (dSTORM) method utilizes cyanine dyes (Cy5 and Alexa 647) switched on by adding a special buffer containing thiol and glucose oxidase [50]; a wide range of fluorescent labels for SMLM have been described in refs. [51, 52]. Another SMLM technique, the point accumulation for imaging in nanoscale topography (PAINT) method, uses diffusible dyes that are switched on only when being transiently bound to the target structure [53, 54].

**Fig. 6:**
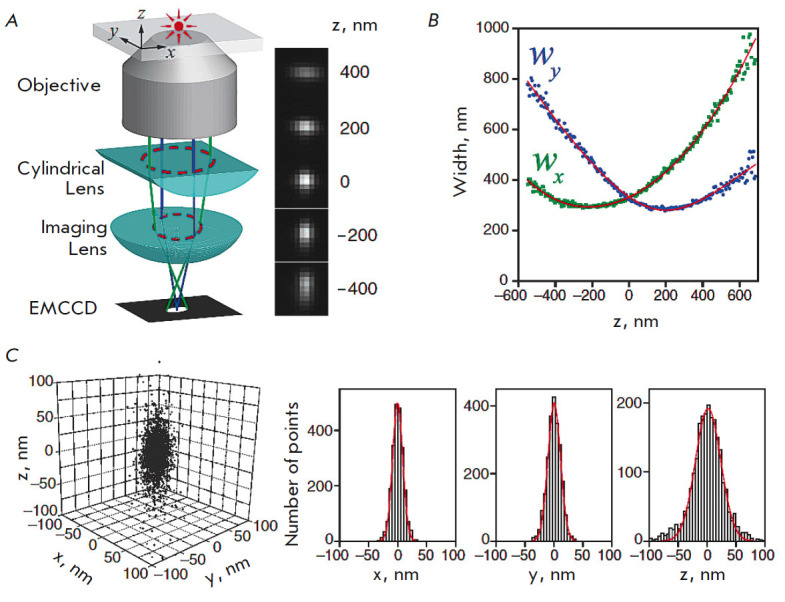
The principle of the 3D STORM technique. (*A*) An optical scheme for determining the axial coordinate of a radiating object by analyzing the ellipticity of its image. The right panel shows images of the radiating object in the X and Y planes depending on its axial position. (*B*) Examples of the dependence of the ellipticity of the image of the emitting object (Alexa 647) for X and Y coordinates on focusing along the Z axis. (*C*) An example of the 3D distribution of single emitting objects and the corresponding histograms of the distribution in the X, Y, and Z directions. The figure was taken from ref. [45]

Elaboration of the STORM-based 3D-SMLM method is based on allowance for the astigmatism of the images obtained at different specimen depths [55]. It is achieved by using a special cylindrical lens with a small radius of curvature in the optical scheme so that focal planes differ for the X and Y directions. The fluorophore occurrence depth is calculated according to changes in the ellipticity of the spatial distribution of its emission. The operating principle of this approach is shown in *Fig. 6*. Thus, *Fig. 6B* illustrates the difference in the ellipticity minima for the X and Y coordinates and the method for determining the true specimen position on the Z coordinate according to the intersection of the X and Y ellipticity dependences. *Figure 6C* also shows an example of 3D localization distribution of single molecules (in this case, Alexa 647 fluorophore). The experimentally observed cluster of localizations (intersections of ellipticity curves) of the same molecule is related to its multiple activation/deactivation. Therefore, it is necessary to localize a sufficiently large number of clusters (145 in this case) that are further statistically aligned with respect to the center of mass to obtain the overall three-dimensional distribution of the localizations (left panel in *Fig. 6C*).

**Fig. 7:**
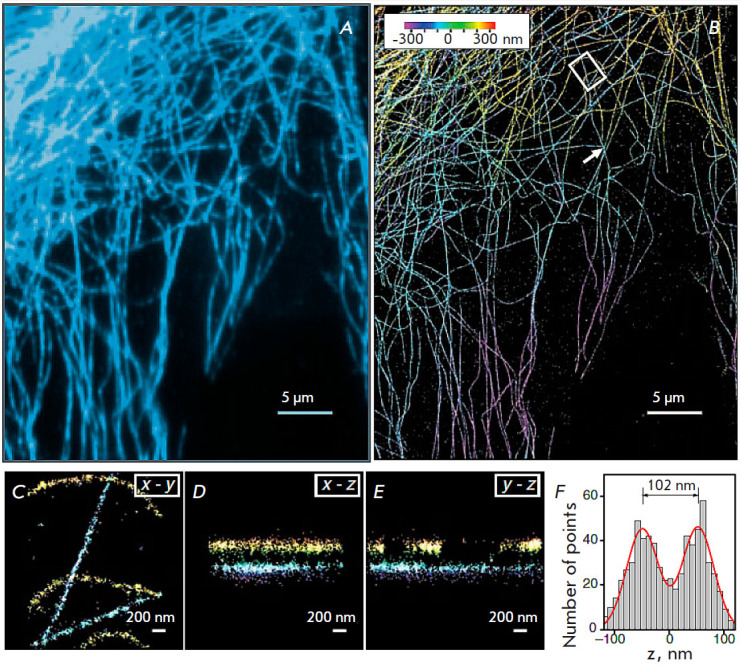
Results obtained using the 3D STORM technique. (*A*) A widefield fluorescent image of microtubules in a BS-C-1 cell. (*B*) An image obtained in the 3D STORM mode of the same section of the BSC-1 cell as that shown in panel (*A*). Data on the axial coordinates of the occurrence are presented in the pseudo-color scale. (*C–E*) The cross sections corresponding to the five microtubule strands in the X-Y, X-Z, and Y-Z directions in the BS-C-1 cell area are shown with a white rectangle in (*B*). (*F*) Z-profile histogram of two microtubules intersecting in the X-Y projection, plotted in the area indicated with a white arrow in panel (*B*). The figure was taken from ref. [45]

Imaging of a BS-C-1 cell stained with primary and secondary antibodies, as well as Cy3 and Alexa 647 dyes, can be reported as an example of a practical application of this technique [56]. The result of using this approach is demonstrated in *Fig. 7*.

Unlike for 3D-SMLM, in the case of 3D optical imaging by STED, the investigated area is illuminated not completely but pointwise using two focused lasers. The first laser scans the surface in a way similar to a conventional confocal microscope, while the beam profile of the second laser in the focal plane is shaped as a torus projection and used to suppress spontaneous emission at the margins of the investigated area, using stimulated emission. A resolution of several tens of nanometers is achieved due to the fact that the recorded radiation originates exactly from the center of the excitation laser beam [57]. The nearzero intensity in the center of the suppressing beam is an essential factor in this case; otherwise, STED efficiency decreases because of a suppression of radiation in the investigated region [58]. Several types of STED can be differentiated depending on the light distribution in the suppressing beam: 1D-STED (X or Y directions) [59], 2D-STED (X and Y simultaneously) [60], z-STED (along the optical axis Z), and, finally, 3D-STED [61, 62]. The major technical challenge for 3D-STED consists in a noncoherent integration of two suppressing beams, one having a profile identical to that for 2D-STED and the other one identical to that for z-STED. This approach also allows one to separately adjust the axial and lateral resolutions. The shape of the suppressing beam is set by creating certain phase patterns. Fixed phase delay plates or spatial light modulators are used for this purpose.

**Fig. 8:**
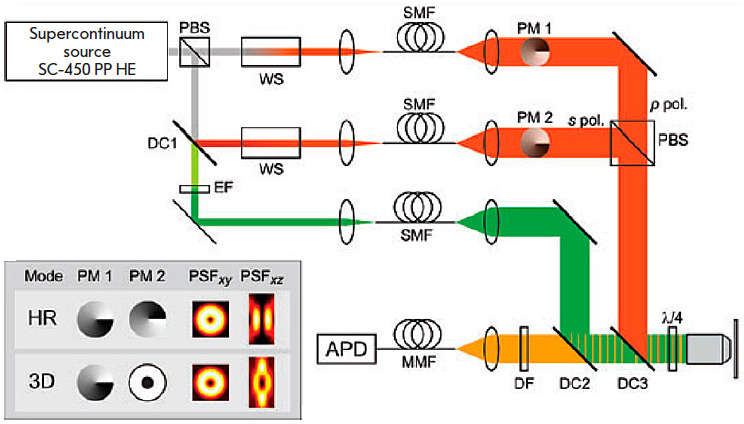
Schematic diagram of the setup for implementing the 3D-STED technique using a supercontinuum laser. The figure was borrowed from ref. [61]

Strictly synchronized pulsed laser sources have conventionally been used in STED microscopy; their operating principle is as follows: a fluorescence-exciting pulse (~ 80 ps, shorter than the fluorescence lifetime) is followed by a STED pulse with a duration of ~ 250 ps, narrowing the spatial region. However, extension of STED microscopy to the visible spectrum necessitated the use of sophisticated nonlinear optics for pulse shaping and synchronization. It has been shown recently [63] that STED microscopy can be conducted using supercontinuum lasers, which greatly simplifies the hardware assemblies for STED microscopy and may contribute to wider implementation of this technique. *Figure 8* shows a schematic diagram of the 3D-STED setup on a supercontinuum laser.

**Fig. 9:**
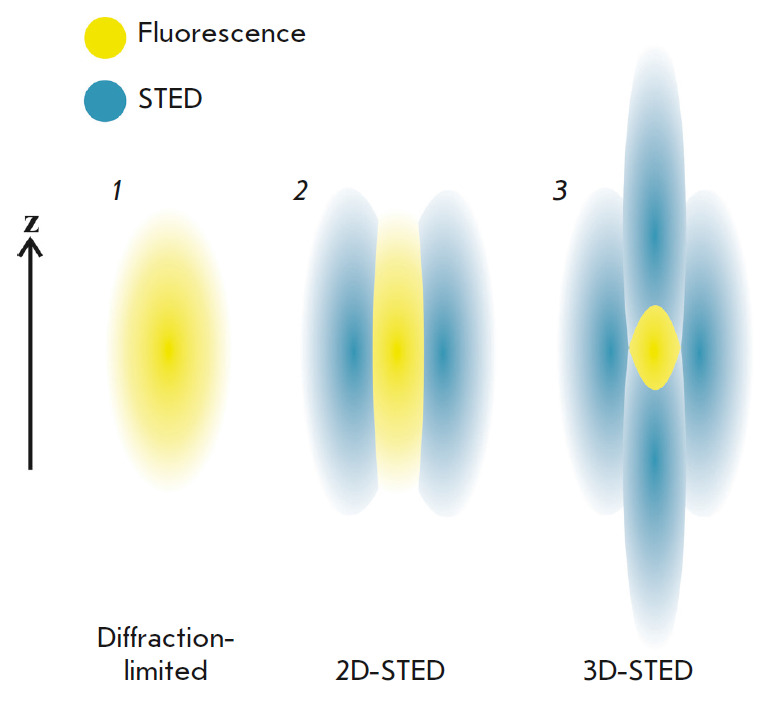
Narrowing of the effective radiation area due to STED suppression of peripheral radiation. *1 *– diffractionlimited fluorescent PSF. *2 *– the torus-shaped STED beam narrows the diameter of the fluorescent PSF. *3 *– fluorescent PSF in the case of 3D-STED

A polarizing beam splitter (PBS) divides laser radiation into two orthogonally (*s *and *p*) polarized beams. The required wavelength is extracted from each beam using a wave selector (WS), and the beam is spatially filtered by single-mode optical fibers (SMFs). The profile of suppressing beams (highlighted in red in the figure) is then set by phase plates (modulators) (PM1 and PM2), and the final beams are summed up by a polarizing beam splitter and directed to the lens entrance by a dichroic mirror (DC3). Portion *s *of the polarized beam that has passed through the dichroic mirror (DC1) is used as the exciting radiation. The extracting filter (EF) isolates the desired wavelength (highlighted in green in the figure); the beam is then also spatially filtered and directed to the lens entrance by the dichroic mirror (DC2). A quarter-wavelength plate in front of the lens makes all the incoming beams circularly polarized. The Stokes fluorescence signal (highlighted in yellow in the figure) passes through both dichroic mirrors, is filtered from the exciting radiation by an optical (dichroic) filter (DF), is focused into a multi-mode optical fiber (MMF) acting as a confocal aperture, and detected by an avalanche photodiode (APD). Scanning takes place as the sample is moved with a three-axis piezo positioner. *Figure 8* also shows the point spread functions (PSFs) in multiple directions when using phase plates for ultrahigh lateral resolution (HR) and acquiring 3D images [64]. Details of the narrowing of the effective radiation area for 3D-STED are shown in *Fig. 9*.

**Fig. 10:**
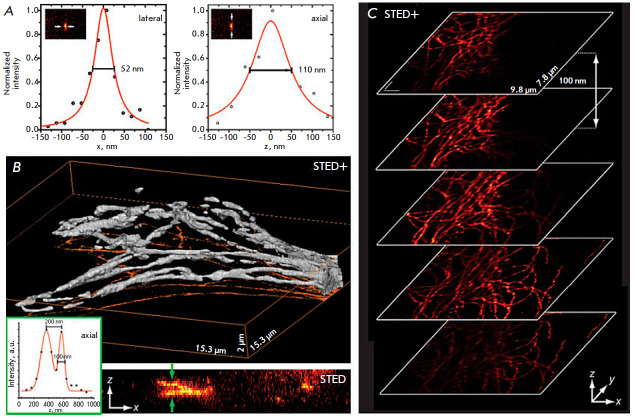
The results obtained using the 3D-STED technique. (*A*) Spatial resolution of fluorescent nanoparticles 44 nm in diameter: 52 nm in the X-direction and 110 nm in the axial (Z) direction. (*B*) A 3D image of fluorescently labeled microtubules: visualization of the isosurface (top) and projection of maximum intensity along the Y axis (bottom) – 30 sections. The inset shows the intensity profile at the specified location. (*C*) Sections obtained at a 100 nm increment in the Z direction. The figure was taken from ref. [61]

Laser intensity and optical aberrations are the key parameters affecting the spatial resolution of STED. For biological specimens, this value is several tens of nanometers. Work is currently underway aiming to eliminate aberrations arising from the use of longfocus lenses and optical windows, which have enabled aberration-free 3D images with a depth of tens of nanometers [65]. *Figure 10* shows an example of the operation of 3D-STED.

An important feature of the method is that nanoscale resolution is achieved without mathematical signal processing. However, studies focusing on combining STED and SMLM are currently underway; an example is the 3D-MINFLUX (minimal photon fluxes) technique with a resolution of several nanometers, which is record-breaking for fluorescence microscopy [66, 67].

## SPM-BASED ULTRASTRUCTURAL 3D MICROSCOPY METHODS

**Fig. 11:**
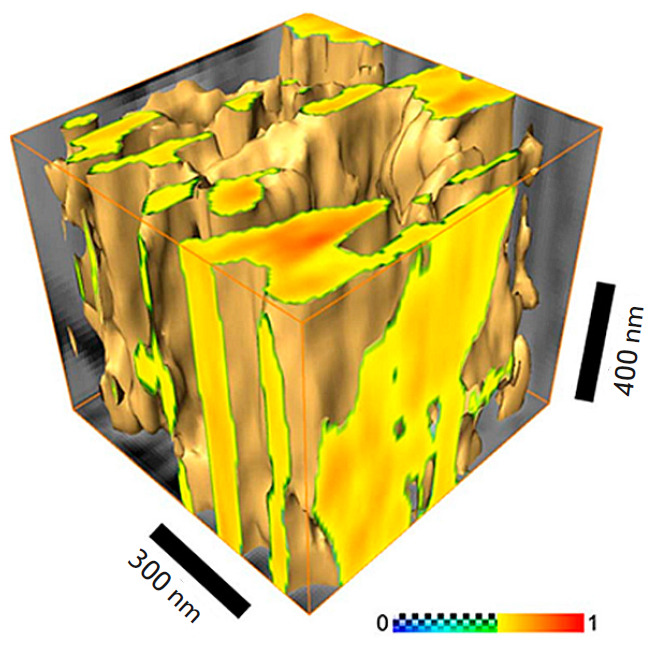
Tomographic reconstruction of a section of human bone, 256 × 256 × 19 voxels, step-by-step chemical etching of 80 nm. The color scale is the value of the phase shift of the SPM probe oscillations normalized to unity. The figure is borrowed from ref. [70]

The approach to 3D analysis based on scanning probe microscopy is in many respects similar to another surface mapping method described previously, scanning electron microscopy, and requires removing the scanned nanolayer from the material surface. The principle of 3D reconstruction is the same as that for FIB-SEM: merging 2D SPM images into a 3D one. The most essential difference between SPM and EM consists in the slightly lower average lateral (XY) spatial resolution that depends on the probe’s radius of curvature. The radius of curvature of standard SPM probes specified by the manufacturer is ~ 10 nm; however, there are quite a few specialized probes (e.g., those with grown diamond whiskers (https://tipsnano.ru/catalog/afm-special/super-sharp/ nsg10-dlc/) whose radius of curvature is ~ 1 nm). SPM was first applied when performing a 3D study of the microdomains of the polystyrene-block-butadiene- block-styrene-type triblock copolymer [68]. Controlled plasma etching was used to remove the layers in this case, which allowed for the removal of 7.5 nm of the material after each etching run. As a result, a 200 × 160 × 45 nm area was reconstructed. A significant drawback of this method was that the mode (*Fig. 11*). The image was obtained after acquiring 19 scans in the phase-contrast imaging mode with etching in hydrochloric acid.

The most significant shortcoming of this method is related to etching, the process used to remove the material, making it impossible to study porous materials, as well as nanocomposites (for them, different reaction rates of the components cause distortions during surface scanning and, therefore, incorrect volume reconstruction). This drawback can be partially compensated for by using special data processing algorithms [70], but the final resolution will differ for some areas, having a negative effect on the analysis of the results.

Using an ultramicrotome is another approach to 3D SPM [73, 74]. The minimal section thickness depends on the ultramicrotome characteristics and is 20 nm for modern setups. It does not matter how the sample was immobilized: using polymer resin or by cryofreezing.

**Fig. 12:**
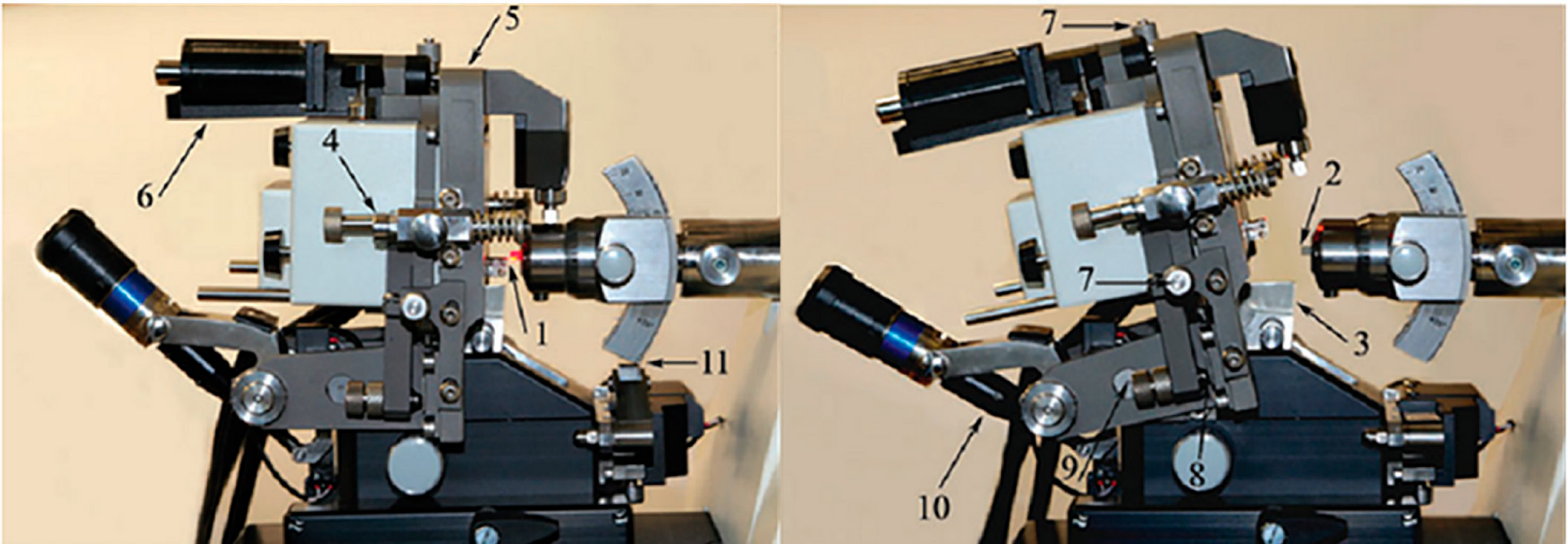
A setup for implementing the SPM-UMT procedure: Ntegra Tomo unification methodology (NT-MDT, Russia). The left panel is the working position for carrying out SPM measurements; the right panel – the SPM head is reserved for performing the UMT cut. (1) SPM probe holder; (2) test specimen; (3) UMT knife holder; (4) SPM-head supports; (5) SPM-head support platform; (6) SPM-head motorized supply system; (7) micrometer screws of the SPM-head positioner; (8) polycorundum support plates; (9) SPM-head hinge fastening system; (10) the system of motorized removal of the SPM-head to bring it to the position of the UMT-cut; and (11) The restrictive support of the UMT console. The figure was taken from ref. [73]

A device for conducting SPNT is shown in *Fig. 12*. It consists of the SPM scanning head attached by special hinges to the UTM knife holder. The design allows for two positions: for scanning (*Fig. 12, right*) and for measurements (*Fig. 12, left*). In the former position, the scanning head is moved away from the movable UTM console, which in turn moves to cut a section. In the latter position, the UTM console returns to its original state, the SPM is brought in, and the remaining specimen portion is scanned [73]. A similar approach has previously been described for SEM; it prevents mechanical distortions of the scanned object such as compression, stretching, and deformation.

The acquired series of successive SPM images are used for the reconstruction and imaging of 3D nanostructures in the bulk of the studied specimens. Application of different SPM measurement techniques allows one to collect information on morphology, as well as local electrical [75], mechanical [76], and many other properties. The technique enables reconstruction of the 3D distribution of nanoparticles in the bulk of nanomaterials [75, 76] and 3D topology of nanoporous structures [77, 78, 7, 80].

In particular, the SPNT method can be efficiently used for 3D reconstruction of micro- and nanofiber cellular scaffolds based on biopolymers [81, 82] and determining their volumetric porosity, surface area to volume ratio, and other 3D morphology parameters. Reconstruction of the 3D structures of cell-engineered constructs allows one to study the topology and numerical morphological parameters of cells and cell– scaffold interfaces, which can act as indicators of the state and biological activity of the cells [83, 84].

**Fig. 13:**
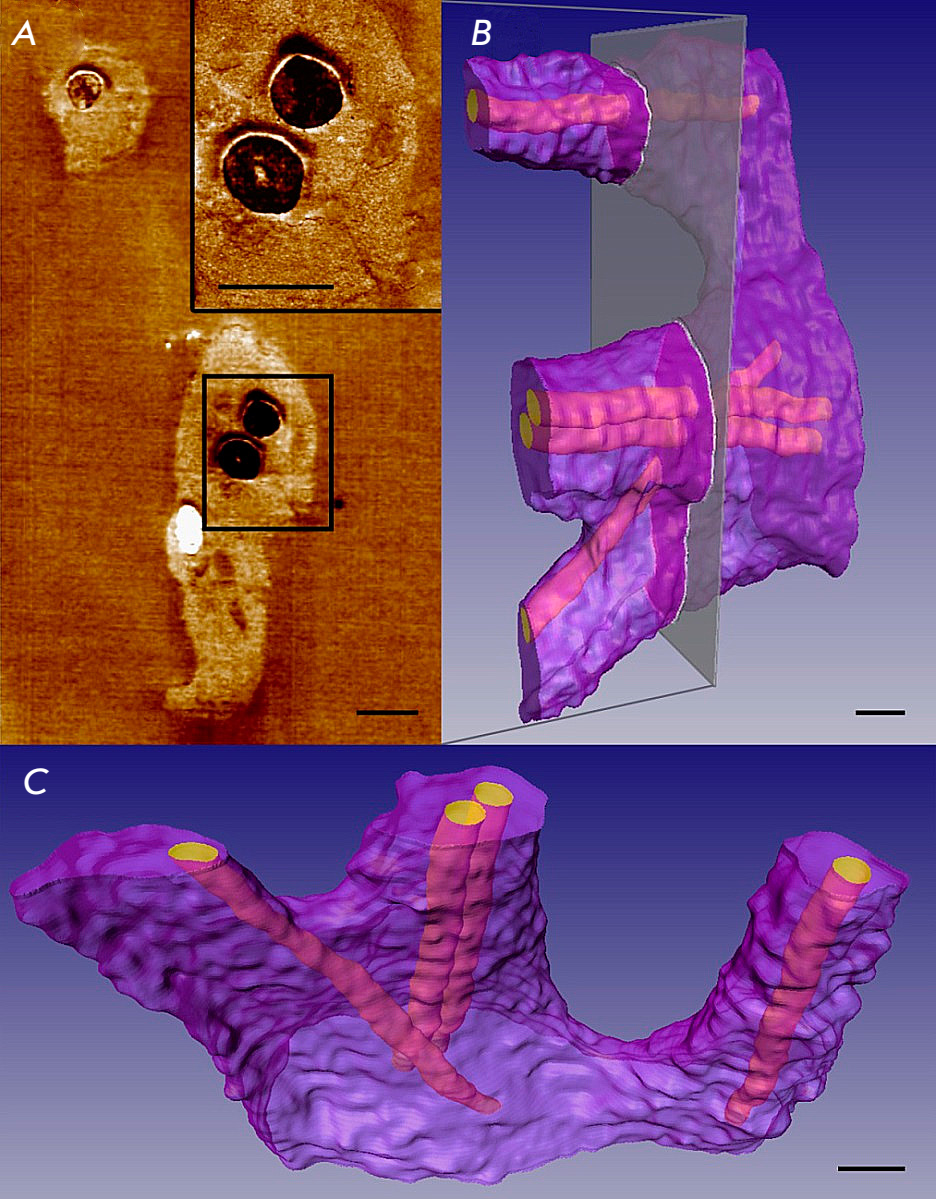
3D SPNT reconstruction of a cardiomyocyte enveloping nanofibers. (*A*) One of the topographic SPM images (phase contrast) used for 3D reconstruction. Insert: a zoomed-in area, shown with a rectangle, including fibers and a membrane fold; (*B, C*) 3D models of a cardiomyocyte enveloping nanofibers (16.0 × 16.0 × 6.5 μm, 54 sections, 120 nm section thickness). The selected plane in (*B*) corresponds to the position of the SPM image in (*A*). The dimensional bar is 1 μm. The figure was taken from ref. [83]

Thus, the characteristic features of the interaction between neonatal rat cardiomyocytes and polymeric nanofiber matrices were revealed by SPNT: it was found that cardiomyocytes, unlike fibroblasts, completely envelop nanofibers in most cases, thus significantly increasing the area of the cell–fiber contact zone. *Figure 13* shows a 3D SPNT reconstruction of the region of a cardiomyocyte enveloping suspended polylactide nanofibers. A total of 54 segmented SPM images of the specimen surface were used for this 3D reconstruction. Each image was acquired sequentially after each UTM cut 120 nm thick [83].

**Fig. 14:**
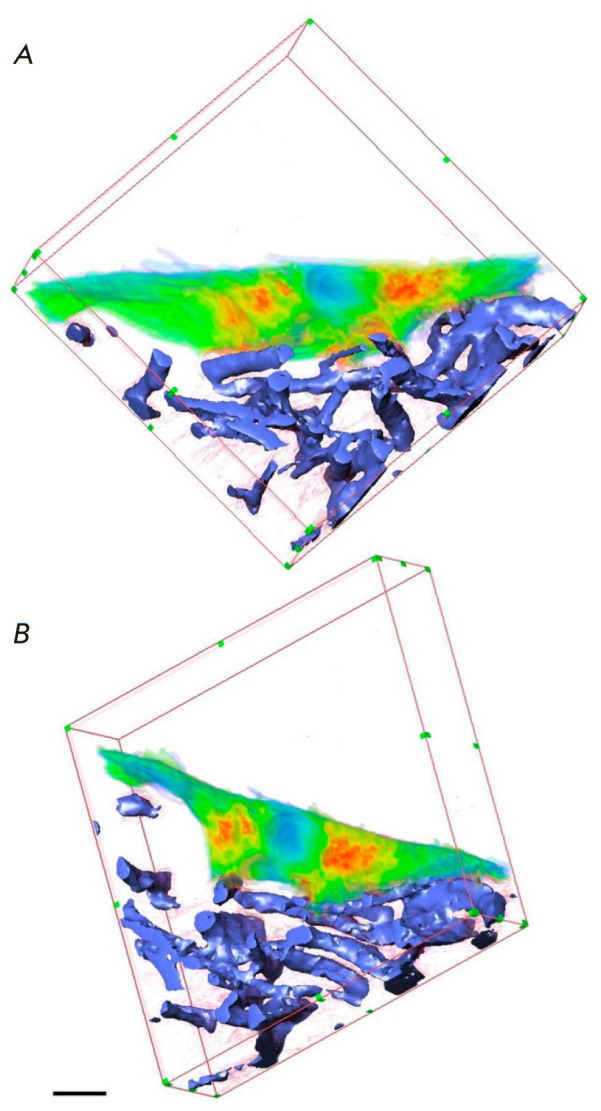
Visualization of the 3D SPNT reconstruction of a fibroblast fragment (shown with green and red) and surrounding polyurethane fibers (blue), 23 sections 150 nm thick, reconstructed volume 32.0 × 32.0 × 3.3 μm, scale bar is 3 μm. The reconstructed fibroblast fragment is shown in two views (*A *and *B*). The measurements were carried out in the phase contrast mode under normal atmospheric conditions at room temperature. The figure was taken from ref. [84]

Another example is the 3D structure of a fibroblast fragment shown in *Fig. 14* (primary human fibroblast culture) contacting several fibers of the microfibrillar polyurethane matrix to form the typical cell membrane protrusions partially enveloping the fibers [84].

**Fig. 15:**
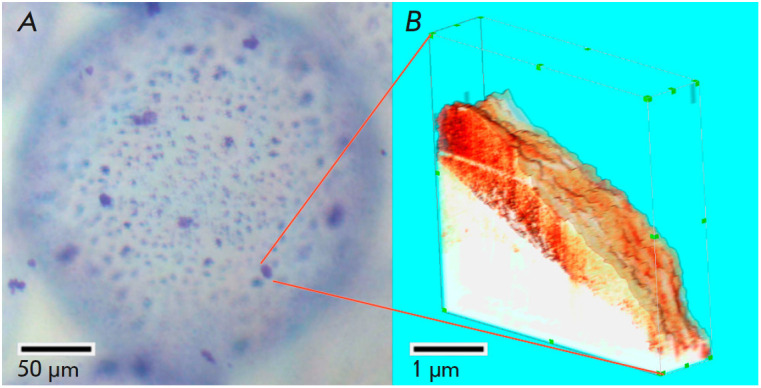
Cryo-SPNT reconstruction of a single microparticle of the rat liver extracellular matrix on the surface of an alginate microcarrier performed at –120°C: (*A*) optical microscopy, Coomassie Brilliant Blue R-250 staining; (*B*) 3D cryo-SPM reconstruction of a single rat liver extracellular matrix microparticle obtained from 13 sequential cryo-SPM images of the microparticle surface on a spherical alginate microcarrier after successive 80-nm thick cryosections. The reconstructed volume is 5.0 × 5.0 × 1.1 μm. The resolution of each 2D SPM scan is 400 × 400 pixels. The pseudocolor palette corresponds to the phase shift of the SPM probe oscillations normalized to unity. The figure was taken from ref. [87]

In order to study soft biopolymeric materials and biological objects without embedding them into an epoxy medium, they need to be preliminarily frozen; for this purpose, we have designed a setup combining SPM and a cryo-ultramicrotome chamber [85]. This setup allows one to perform successive SPM measurements on the surface of frozen specimens immediately after cutting with a diamond cryo-ultramicrotome knife in the cryochamber. In this case, the measurements are performed in the semi-contact mode using cantilevers mounted on quartz resonators and not requiring an optical deflectometer to be used, which is important for working with a cryochamber. *Figure 15* shows the 3D cryo-SPNT reconstruction of a single microparticle of a rat liver extracellular matrix on the surface of an alginatemicrocarrier shown as an example of the operation of this setup performed at –120°C, since sectioning at higher temperatures disrupts the structure of hydrogel microcarriers [86, 87].

A detailed protocol for obtaining specimens of rat liver extracellular matrix microparticles was reported in ref. [86]. Using this protocol, cells were completely removed from the extracellular matrix, whose fragments were comminuted in liquid nitrogen; this procedure yielded extracellular matrix microparticles 1–5 μm in size. The resulting microparticles were then covalently crosslinked to alginate microspheres 200–300 μm in size (*Fig. 15A*).

Capabilities in analyzing the 3D nanostructures of biological objects can be substantially broadened by combining SPNT and high-resolution optical microscopy techniques, and fluorescence microscopy in particular, into a single correlative optical probe nanotomography technique [88, 89, 91], which can be implemented using a unique research setup (http://ckp-rf. ru/usu/486825/).

**Fig. 16:**
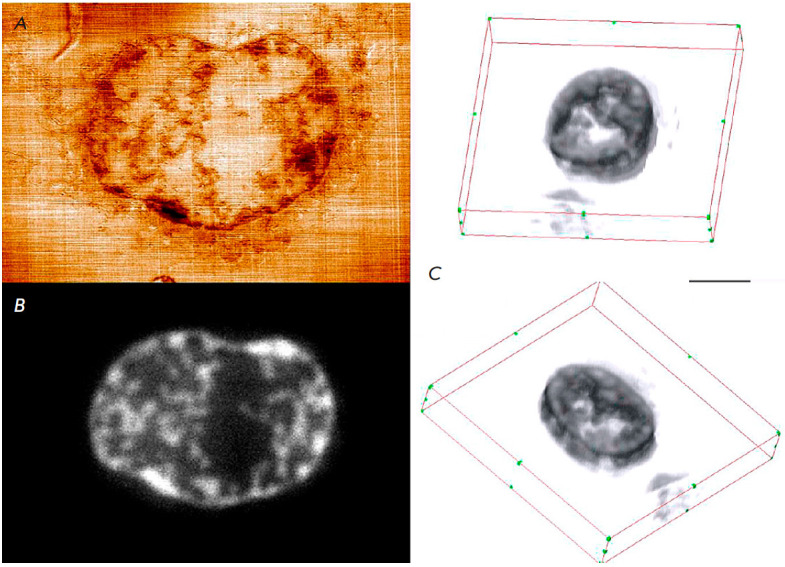
Analysis of human breast adenocarcinoma MCF-7 cell samples with doxorubicin. (*A*) An SPM image of the topography of the cut surface of the MCF-7 cell, scan size 13.8 × 9.5 μm; height variation range, 33.5 nm; (*B*) fluorescent image of a cut of the same area of the MCF-7 cell; (*C*) 3D reconstruction of doxorubicin distribution in the volume of the MCF-7 cell sample, 22.5 × 18.7 × 2.4 μm; section thickness, 120 nm; the dimensional segment, 5 μm; visualization is presented in two views. The figure was taken from ref. [92]

*Figure 16* shows an example of correlative fluorescence and SPM images of an MSF-7 cell and visualization of the 3D reconstruction of the doxorubicin distribution in the cell based on the data obtained. The 3D reconstruction shows a coherent distribution structure that can be used as a successful measure of the employed 3D method. Resolution of the reconstruction along the Z coordinate (in the axial direction) corresponds to the section thickness (120 nm) in this case [92].

## CONCLUSIONS

Among the 3D structural reconstruction methods discussed in this review, one cannot be singled out, as the best since each of these techniques has its own advantages and shortcomings.

The key advantage of electron microscopy-based methods is the very high spatial resolution ( < 1 nm ) and the possibility of acquiring images from significant depths thanks to the layer-by-layer scanning mode; however, the use of vacuum, as well as the electron and ion beams in them, can damage the specimen and alter the native structures of polymers and protein compounds. Furthermore, these methods provide no information other than sample morphology.

The STORM and STED optical methods have a lower spatial resolution (several nanometers), but they allow one to both conduct an ultrastructural analysis of a specimen and reconstruct the spatial distribution of target objects thanks to highly specific fluorescent immunostaining. Meanwhile, significant limitations are imposed on the size of the area being analyzed. Aberrations must be eliminated to obtain high-quality images, making it necessary to use complex optical systems.

Similar to EM, the application of scanning probe microscopes for 3D reconstruction allows one to reconstruct images of specimens with a large depth by removing some material, as well as obtain information on the chemical structure, electrical, and magnetic properties using the phase contrast method and special conductive, magnetic, or functionalized probes. Meanwhile, this technique is inferior to electron and optical microscopy in terms of lateral resolution.

Simultaneous use of SPM correlative measurement techniques and high-resolution fluorescence microscopy for the 3D reconstruction of the ultrastructure of biological objects is promising for enhancing the information value of the 3D data obtained (in particular, the 3D distributions of fluorescent markers and nanoscale morphological features).

## Abbreviations

EM: electron microscopy
SEM: scanning electron microscopy
TEM: transmission electron microscopy
OM: optical microscopy
SPM: scanning probe microscopy
STORM: stochastic optical reconstruction microscopy
STED: stimulated emission depletion
ssTEM: serial section transmission electron microscopy
AT: array tomography
ATUM-SEM: automated tape collecting ultramicrotome scanning electron microscopy
SBF-SEM: serial block-face scanning electron microscopy
FIB-SEM: focused ion beam scanning electron microscopy
ET: electron tomography
ECT: electron cryotomography
STA: subtomogram averaging
AECs: alveolar epithelial cells
SMLM: single-molecule localization microscopy
PBS: polarizing beam splitter
WS: wave selector
PM: phase modulator
SMF: single-mode optical fiber
DC: dichroic mirror
DF: dichroic filter
EF: extracting filter
MMF: multi-mode optical fiber
APD: avalanche photodiode
EMCCD: electron multiplying charge-coupled device
PALM: photo-activated light microscopy
PAINT: point accumulation for imaging in nanoscale topography
MINFLUX: minimal photon fluxes
SPNT: scanning probe nanotomography
PSF: point spread function
